# Supplementary figures and images for: Association between surgeon training grade and the risk of revision following total knee replacement: An analysis of National Joint Registry data
Source: PLoS Med. 2025 Aug 12;22(8):e1004685. doi: 10.1371/journal.pmed.1004685 (PMC12370202; doi:10.1371/journal.pmed.1004685)

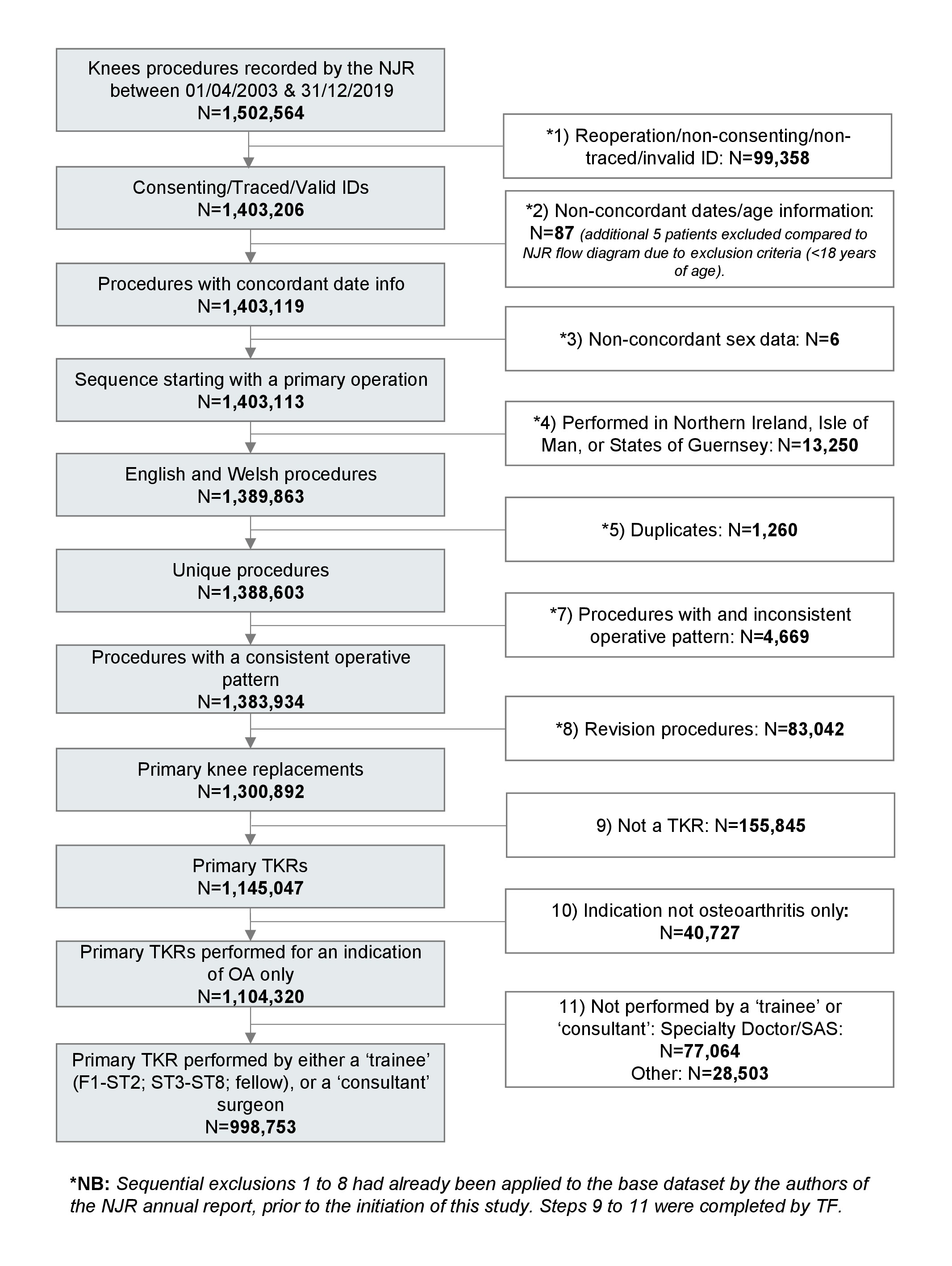

Supplement: S1 Fig — (TIFF) [file pmed.1004685.s001.tiff]

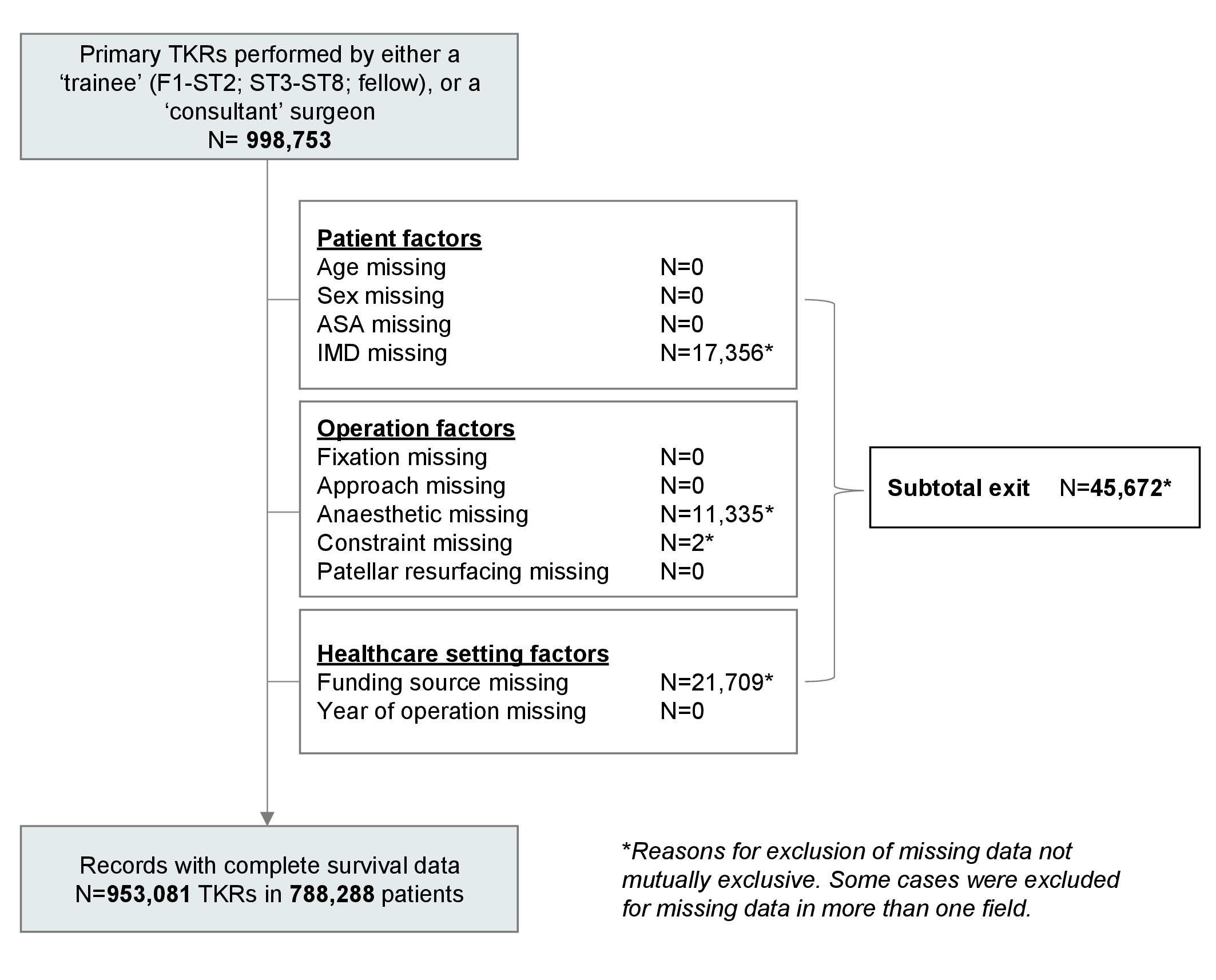

Supplement: S2 Fig — (TIFF) [file pmed.1004685.s002.tiff]

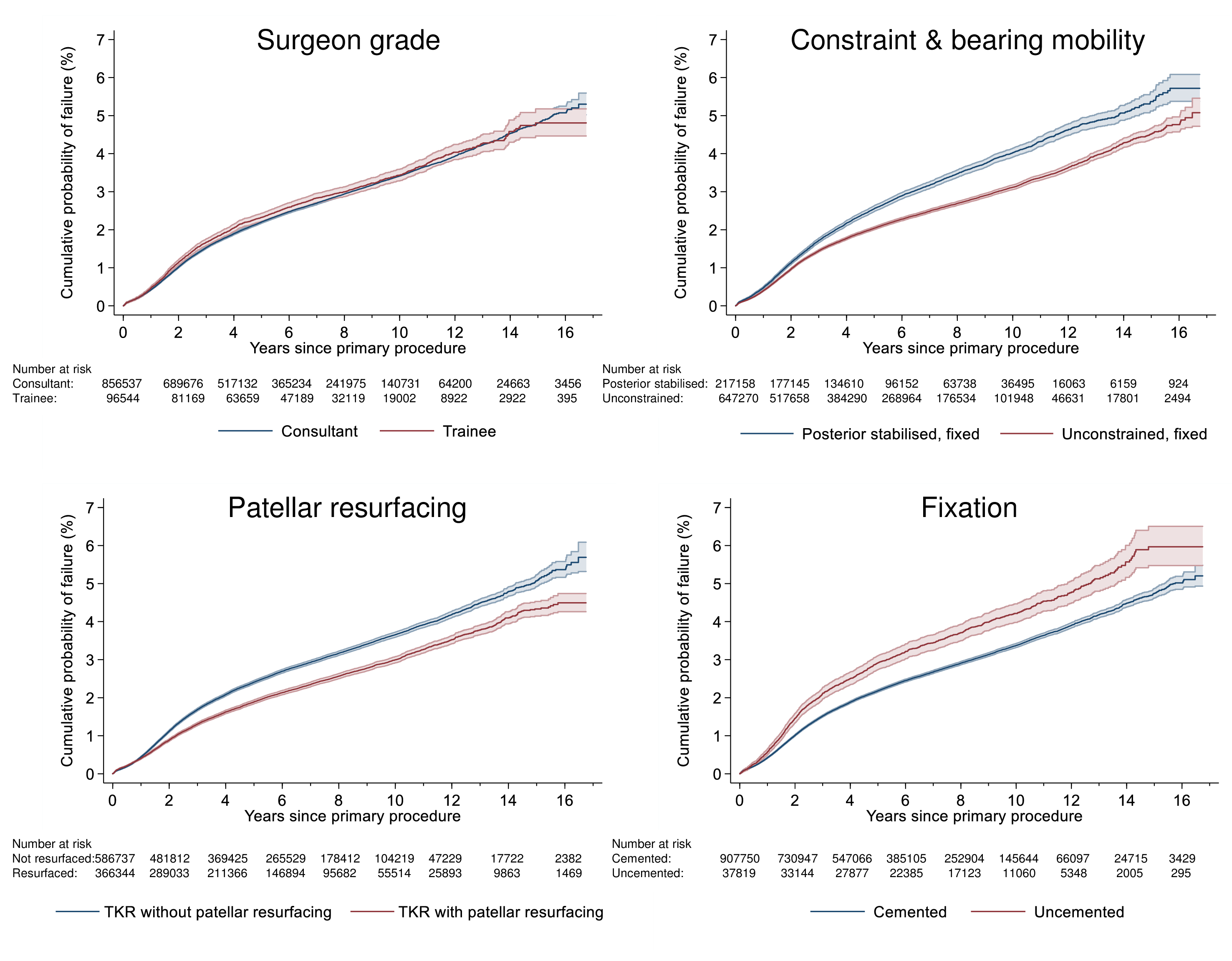

Supplement: S3 Fig — (TIFF) [file pmed.1004685.s003.tiff]
