# Supplementary material for: Association between surgeon training grade and the risk of revision following total knee replacement: An analysis of National Joint Registry data
Source: PLoS Med. 2025 Aug 12;22(8):e1004685. doi: 10.1371/journal.pmed.1004685 (PMC12370202; doi:10.1371/journal.pmed.1004685)
Supplement: S1 Appendix — (DOCX) [file pmed.1004685.s004.docx]

S1 Appendix - Process of accounting for changes in NJR operating surgeon grade categories.

The current NJR MDS form (version 7.0) uses the following ‘operating surgeon grade’ categories: Consultant; SPR/ST3-ST8; F1-ST2; Specialty Doctor/SAS; and Other. However, since April 2003 there have been seven different versions of the MDS form, and the ‘operating surgeon grade’ categories have been updated with each iteration to reflect current training terminology. The following table details the process by which these variations have been accounted for, e.g. by recoding NJR records of ‘operating surgeon grade’ to correspond to the current MDS 7.0 categories.

| **NJR record of operating surgeon grade**  *(Based on numerous previous MDS versions)* | **Recoded to MDS 7.0 operating surgeon grade categories** | **Surgeon grade (binary variable)** |
| --- | --- | --- |
| Consultant | Consultant | Consultant |
| SpR/ST3-ST8 | SpR/ST3-ST8 | Trainee |
| SPR |  |  |
| F1-ST2 | F1-ST2 |  |
| House Officer (HO) |  |  |
| Senior House Officer (SHO) |  |  |
| Fellow | Other |  |
| Other |  | Not included in primary analyses |
| Visiting Overseas Specialist |  |  |
| Specialty Doctor/SAS | Specialty Doctor/SAS |  |
| Associate Specialist |  |  |
| Staff grade |  |  |
