# Supplementary material for: Association between surgeon training grade and the risk of revision following total knee replacement: An analysis of National Joint Registry data
Source: PLoS Med. 2025 Aug 12;22(8):e1004685. doi: 10.1371/journal.pmed.1004685 (PMC12370202; doi:10.1371/journal.pmed.1004685)

**S2 Appendix - Model Schematic summary of surgical training in the UK.**

Schematic overview of surgical training in the UK. Adapted from: Fitzgerald JEF et al. (2012).


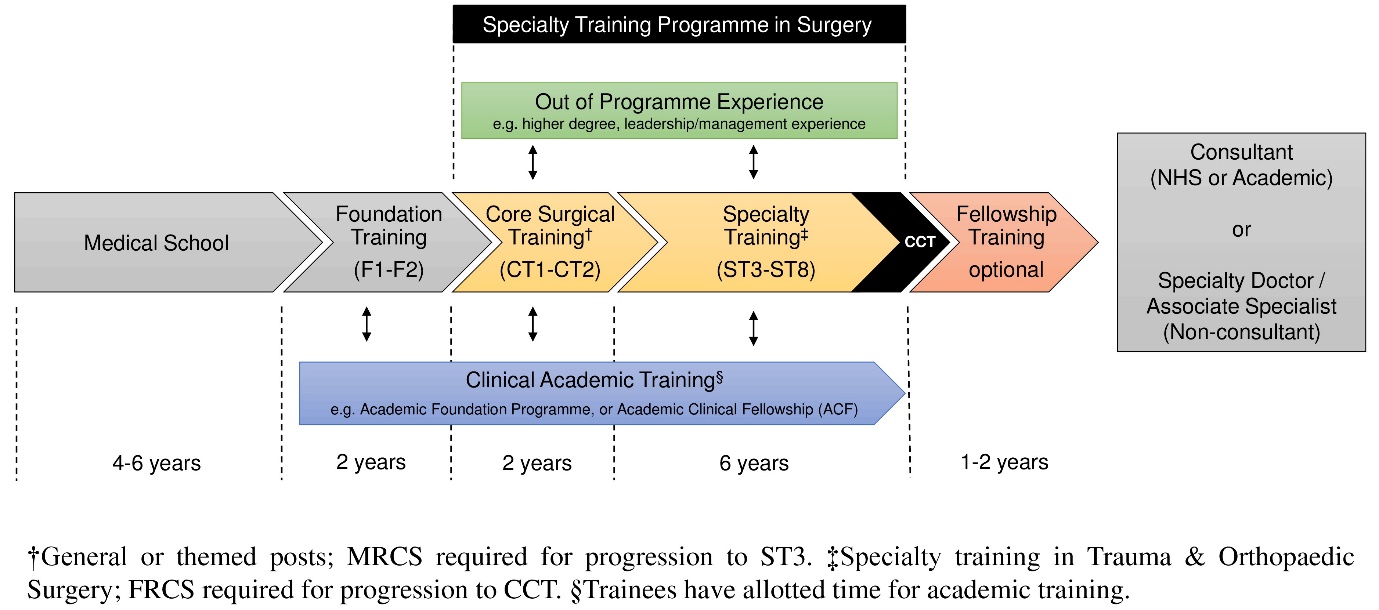

Supplement: S2 Appendix — (DOCX) [file pmed.1004685.s005.docx]
