# Supplementary material for: Association between surgeon training grade and the risk of revision following total knee replacement: An analysis of National Joint Registry data
Source: PLoS Med. 2025 Aug 12;22(8):e1004685. doi: 10.1371/journal.pmed.1004685 (PMC12370202; doi:10.1371/journal.pmed.1004685)
Supplement: S3 Appendix — (DOCX) [file pmed.1004685.s006.docx]

S3 Appendix – Model selection, construction, and justification.

The structured approach to model selection and construction used in this study was based around an in-depth assessment of the proportionality of hazard functions. Where data did not satisfy the PH assumption (i.e. the ratio of hazard functions was not constant) and further analysis demonstrated that FPM was superior to the Cox model, FPM was preferred for primary analysis. This approach is summarised here:

**Step 1:** We performed an initial analysis using Cox regression. Schoenfeld residuals plots/tests were applied to incrementally adjusted Cox models for ‘surgeon grade’ to assess the PH assumption.

1. Cox PH model for surgeon grade (Model 1: unadjusted)
   1. Schoenfeld residual test: p=0.005
2. Cox PH model for surgeon grade (Model 2: adjusted for patient factors)
   1. Schoenfeld residual test: p<0.001
3. Cox PH model for surgeon grade (Model 3: adjusted for patient & operation factors)
   1. Schoenfeld residual test: p<0.001
4. Cox PH model for surgeon grade (Model 4: adjusted for patient, operation & healthcare factors)
   1. Schoenfeld residual test: p<0.001

The Schoenfeld residuals tests suggested that ‘surgeon grade’ may have a time-dependent effect. This was supported by the results of likelihood ratio testing, in which PH and non-PH models for ‘surgeon grade’ were compared at each level of adjustment. FPM was used to investigate this further.

**Step 2:** FPMs were constructed according to the methods described by Royston and Lambert [16]. We constructed the following two models:

1. A non-proportional hazards FPM (Model 4: adjusted for patient, operation & healthcare factors)
   1. A non-PH model using the stpm2 command in Stata, with ‘surgeon grade’ specified as having a time-dependent effect.
   2. Graphical assessment, Akaike information criteria (AIC), and Bayes information criteria (BIC) were used to optimise the fit and complexity of the model. These methods were used to determine the degrees of freedom (DF) with which to model hazard functions, as well as the optimal number and location of knots.
   3. We confirmed the superiority of our final model to preceding iterations using likelihood ratio testing.
   4. The final model was as follows: The baseline hazard was modelled with 8 degrees of freedom. Surgeon grade had a time-dependent effect (DFTVE) and was modelled with 2 degrees of freedom. Confounding variables were modelled with fixed effects.

**AIC and BIC details for degrees of freedom (DF)**

| **Model (DF)** | **AIC** | **BIC** |
| --- | --- | --- |
| DF1 | 248677.2 | 248747.8 |
| DF2 | 248247.9 | 248330.2 |
| DF3 | 247866.9 | 247961.0 |
| DF4 | 247352.2 | 247458.1 |
| DF5 | 247345.3 | 247463.0 |
| DF6 | 247327.0 | 247456.4 |
| DF7 | 247286.5 | 247427.7 |
| *DF8** | *247256.1* | *247409.0* |
| **Selected model component* | | |

**AIC and BIC details for degrees of freedom for time-varying effects (DFTVE):**

| **Model (DFTVE)** | **AIC** | **BIC** |
| --- | --- | --- |
| DFTVE1 | 247258.8 | 247388.2 |
| *DFTVE2** | *247254.0* | *247395.2* |
| DFTVE3 | 247256.1 | 247409.0 |
| DFTVE4 | 247257.1 | 247421.8 |
| DFTVE5 | 247258.1 | 247434.6 |
| **Selected model component* | | |

1. A proportional hazards FPM (Model 4: adjusted for patient, operation & healthcare factors)
   1. A PH model using the stpm2 command, equivalent to the Cox model.
   2. This model is identical to the non-PH FPM model described above, but surgeon grade is not specified as having a time-dependent effect.

**Step 3:** Likelihood ratio tests were used to compare PH and non-PH models at each level of adjustment:

1. PH FPM compared to non-PH FPM (Model 1): p=0.003
2. PH FPM compared to non-PH FPM (Model 2): p=0.003
3. PH FPM compared to non-PH FPM (Model 3): p=0.006
4. PH FPM compared to non-PH FPM (Model 4): p=0.006

**Summary:** Non-PH FPMs were superior to PH (i.e. Cox) models in this context, as PH models did not adequately account for time-dependent effects. Therefore, FPM was used as the primary method of adjusted analysis.
