# Supplementary material for: Association between surgeon training grade and the risk of revision following total knee replacement: An analysis of National Joint Registry data
Source: PLoS Med. 2025 Aug 12;22(8):e1004685. doi: 10.1371/journal.pmed.1004685 (PMC12370202; doi:10.1371/journal.pmed.1004685)
Supplement: S4 Appendix — (DOCX) [file pmed.1004685.s007.docx]

S4 Appendix - Model specification summarising the exposures and confounding variables used in the analyses.

| **Model** | **Exposure (surgeon groups)** | **Method** | **Confounding variables included in model** |
| --- | --- | --- | --- |
| 1 | 1. **Surgeon grade:** i. Consultant; ii. Trainee (overall) | FPM | Unadjusted |
|  | 1. **Supervision:** i. Consultant; ii. Trainee supervised by a scrubbed consultant; iii. Trainee not supervised by a scrubbed consultant | FPM |  |
|  | 1. **Specific training grade:** i. Consultant; ii. F1-ST2; iii. ST3-ST8; iv. Fellow | FPM |  |
| 2 | 1. **Surgeon grade:** i. Consultant; ii. Trainee (overall) | FPM | Patient factors^†^ |
|  | 1. **Supervision:** i. Consultant; ii. Trainee supervised by a scrubbed consultant; iii. Trainee not supervised by a scrubbed consultant | FPM |  |
|  | 1. **Specific training grade:** i. Consultant; ii. F1-ST2; iii. ST3-ST8; iv. Fellow | FPM |  |
| 3 | 1. **Surgeon grade:** i. Consultant; ii. Trainee (overall) | FPM | Patient factors^†^  Operation factors^‡^ |
|  | 1. **Supervision:** i. Consultant; ii. Trainee supervised by a scrubbed consultant; iii. Trainee not supervised by a scrubbed consultant | FPM |  |
|  | 1. **Specific training grade:** i. Consultant; ii. F1-ST2; iii. ST3-ST8; iv. Fellow | FPM |  |
| 4 | 1. **Surgeon grade:** i. Consultant; ii. Trainee (overall) | FPM | Patient factors^†^  Operation factors^‡^  Healthcare setting factors^§^ |
|  | 1. **Supervision:** i. Consultant; ii. Trainee supervised by a scrubbed consultant; iii. Trainee not supervised by a scrubbed consultant | FPM |  |
|  | 1. **Specific training grade:** i. Consultant; ii. F1-ST2; iii. ST3-ST8; iv. Fellow | FPM |  |
| ^†^**Patient factors:** age (65-75); gender (female); ASA (II); IMD decile (least deprived) | | | |
| ^‡^**Operation factors:** approach (medial parapatellar); mode of fixation (cemented); constraint (unconstrained fixed); anaesthetic (spinal); patellar resurfacing (not resurfaced) | | | |
| ^§^**Healthcare setting factors:** funding (NHS); year of operation (most recent). ***Baseline category in brackets was the most frequently occurring*** | | | |
| **Time-dependent effects:** Surgeon grade was specified as having a time-dependent effect and was modelled with 2 df. Remaining confounding variables were modelled with fixed effects. The baseline hazard was modelled with 8 df. | | | |
