# Supplementary material for: Association between surgeon training grade and the risk of revision following total knee replacement: An analysis of National Joint Registry data
Source: PLoS Med. 2025 Aug 12;22(8):e1004685. doi: 10.1371/journal.pmed.1004685 (PMC12370202; doi:10.1371/journal.pmed.1004685)
Supplement: S5 Appendix — (DOCX) [file pmed.1004685.s008.docx]

**S5 Appendix – Supplementary Results: Hazard ratio plots for Figure 3-7 presented in tabular format.**

| **Figure 3: Risk of all-cause revision of TKRs according to surgeon grade (exposure A). Results from hazard ratio plots presented in tabular format.** | | | |
| --- | --- | --- | --- |
| **Analysis** | **Follow-up (years)** | **HR** | **95% CI** |
| **Model 1 (unadjusted)** | 1 | 1.12 | 1.05, 1.19 |
|  | 3 | 1.04 | 0.99, 1.09 |
|  | 5 | 0.97 | 0.91, 1.03 |
|  | 7 | 0.93 | 0.86, 1.01 |
|  | 10 | 0.92 | 0.84, 1.00 |
|  | 13 | 0.90 | 0.82, 0.99 |
|  | 15 | 0.89 | 0.81, 0.98 |
| **Model 2 (adjusted for †)** | 1 | 1.17 | 1.10, 1.24 |
|  | 3 | 1.09 | 1.04, 1.14 |
|  | 5 | 1.01 | 0.95, 1.08 |
|  | 7 | 0.98 | 0.90, 1.05 |
|  | 10 | 0.96 | 0.88, 1.04 |
|  | 13 | 0.94 | 0.86, 1.03 |
|  | 15 | 0.93 | 0.85, 1.03 |
| **Model 3 (adjusted for †, ‡)** | 1 | 1.17 | 1.10, 1.24 |
|  | 3 | 1.09 | 1.04, 1.15 |
|  | 5 | 1.02 | 0.96, 1.09 |
|  | 7 | 0.99 | 0.91, 1.06 |
|  | 10 | 0.97 | 0.89, 1.06 |
|  | 13 | 0.95 | 0.87, 1.05 |
|  | 15 | 0.95 | 0.86, 1.04 |
| **Model 4 (adjusted for †, ‡, §)** | 1 | 1.16 | 1.09, 1.23 |
|  | 3 | 1.09 | 1.04, 1.14 |
|  | 5 | 1.01 | 0.95, 1.08 |
|  | 7 | 0.98 | 0.91, 1.06 |
|  | 10 | 0.96 | 0.88, 1.05 |
|  | 13 | 0.95 | 0.86, 1.04 |
|  | 15 | 0.94 | 0.85, 1.03 |
| **Sensitivity Analysis BMI (adjusted for †, ‡, §, and BMI)** | 1 | 1.16 | 1.09, 1.23 |
|  | 3 | 1.09 | 1.04, 1.14 |
|  | 5 | 1.01 | 0.95, 1.08 |
|  | 7 | 0.98 | 0.91, 1.06 |
|  | 10 | 0.96 | 0.89, 1.05 |
|  | 13 | 0.95 | 0.86, 1.04 |
|  | 15 | 0.94 | 0.85, 1.04 |
| **Sensitivity Analysis NHS (adjusted for †, ‡, §, but restricted to NHS-funded cases in NHS hospitals)** | 1 | 1.15 | 1.08, 1.22 |
|  | 3 | 1.07 | 1.02, 1.12 |
|  | 5 | 0.99 | 0.93, 1.06 |
|  | 7 | 0.95 | 0.88, 1.03 |
|  | 10 | 0.94 | 0.86, 1.02 |
|  | 13 | 0.92 | 0.84, 1.01 |
|  | 15 | 0.91 | 0.82, 1.01 |
| HR, hazard ratio; CI, confidence interval; ASA, American Society of Anaesthesiologists grade. **^†^Patient factors:** age; sex; ASA; index of multiple deprivation decile. **^‡^Operation factors:** anaesthetic; approach; fixation; constraint; patellar resurfacing. **^§^Healthcare setting factors:** funding; year of operation. | | | |

| **Figure 4: Risk of all-cause revision of TKRs according to the level of supervision of trainees (exposure B). Results from hazard ratio plot presented in tabular format.** | | | |
| --- | --- | --- | --- |
| **Analysis** | **Follow-up (years)** | **HR** | **95% CI** |
| **Model 4** (adjusted for †, ‡, §) | 1 | 1.01 | 0.90, 1.15 |
|  | 3 | 1.01 | 0.92, 1.11 |
|  | 5 | 0.94 | 0.83, 1.07 |
|  | 7 | 0.91 | 0.79, 1.05 |
|  | 10 | 0.88 | 0.75, 1.04 |
|  | 13 | 0.87 | 0.72, 1.04 |
|  | 15 | 0.86 | 0.71, 1.04 |
| HR, hazard ratio; CI, confidence interval; ASA, American Society of Anaesthesiologists grade. **^†^Patient factors:** age; sex; ASA; index of multiple deprivation decile. **^‡^Operation factors:** anaesthetic; approach; fixation; constraint; patellar resurfacing. **^§^Healthcare setting factors:** funding; year of operation. | | | |

| **Figure 5: Risk of all-cause revision of TKRs according to specific training grade (exposure C). Results from hazard ratio plots presented in tabular format.** | | | |
| --- | --- | --- | --- |
| **Analysis** | **Follow-up (years)** | **HR** | **95% CI** |
| **F1-ST2** (Model 4: adjusted for †, ‡, §) | 1 | 1.22 | 0.73, 2.04 |
|  | 3 | 1.14 | 0.73, 1.77 |
|  | 5 | 0.92 | 0.55, 1.54 |
|  | 7 | 0.79 | 0.41, 1.55 |
|  | 10 | 0.73 | 0.35, 1.54 |
|  | 13 | 0.69 | 0.30, 1.57 |
|  | 15 | 0.67 | 0.28, 1.59 |
| **ST3-ST8** (Model 4: adjusted for †, ‡, §) | 1 | 1.17 | 1.10, 1.25 |
|  | 3 | 1.08 | 1.03, 1.14 |
|  | 5 | 0.99 | 0.93, 1.06 |
|  | 7 | 0.96 | 0.88, 1.04 |
|  | 10 | 0.94 | 0.86, 1.03 |
|  | 13 | 0.92 | 0.84, 1.02 |
|  | 15 | 0.91 | 0.82, 1.01 |
| **Fellow** (Model 4: adjusted for †, ‡, §) | 1 | 0.85 | 0.61, 1.19 |
|  | 3 | 1.07 | 0.85, 1.36 |
|  | 5 | 1.21 | 0.99, 1.47 |
|  | 7 | 1.25 | 1.00, 1.56 |
|  | 10 | 1.27 | 0.98, 1.65 |
|  | 13 | 1.30 | 0.96, 1.75 |
|  | 15 | 1.32 | 0.96, 1.83 |
| HR, hazard ratio; CI, confidence interval; ASA, American Society of Anaesthesiologists grade. **^†^Patient factors:** age; sex; ASA; index of multiple deprivation decile. **^‡^Operation factors:** anaesthetic; approach; fixation; constraint; patellar resurfacing. **^§^Healthcare setting factors:** funding; year of operation. | | | |

| **Figure 6:**  **Risk of all-cause revision of TKRs according to the level of supervision of ST3-ST8 trainees. Results from hazard ratio plot presented in tabular format.** | | | |
| --- | --- | --- | --- |
| **Analysis** | **Follow-up (years)** | **HR** | **95% CI** |
| **Model 4 (adjusted for †, ‡, §)** | 1 | 1.01 | 0.90, 1.15 |
|  | 3 | 1.00 | 0.91, 1.10 |
|  | 5 | 0.94 | 0.83, 1.07 |
|  | 7 | 0.90 | 0.73, 1.05 |
|  | 10 | 0.88 | 0.75, 1.04 |
|  | 13 | 0.86 | 0.70, 1.05 |
|  | 15 | 0.85 | 0.69, 1.05 |
| HR, hazard ratio; CI, confidence interval; ASA, American Society of Anaesthesiologists grade. **^†^Patient factors:** age; sex; ASA; index of multiple deprivation decile. **^‡^Operation factors:** anaesthetic; approach; fixation; constraint; patellar resurfacing. **^§^Healthcare setting factors:** funding; year of operation. | | | |

| **Figure 7: The indication for TKR revision according to surgeon grade (exposure A). Results from hazard ratio plots presented in tabular format.** | | | |
| --- | --- | --- | --- |
| **Analysis** | **Follow-up (years)** | **HR** | **95% CI** |
| **All-cause revision (adjusted for †, ‡, §)** | 1 | 1.16 | 1.09, 1.23 |
|  | 3 | 1.09 | 1.04, 1.14 |
|  | 5 | 1.01 | 0.95, 1.08 |
|  | 7 | 0.98 | 0.91, 1.06 |
|  | 10 | 0.96 | 0.88, 1.05 |
|  | 13 | 0.95 | 0.86, 1.04 |
|  | 15 | 0.94 | 0.85, 1.03 |
| **Aseptic loosening/lysis (adjusted for †, ‡, §)** | 1 | 1.19 | 1.04, 1.36 |
|  | 3 | 1.08 | 0.98, 1.19 |
|  | 5 | 1.03 | 0.94, 1.13 |
|  | 7 | 0.98 | 0.86, 1.11 |
|  | 10 | 0.97 | 0.84, 1.12 |
|  | 13 | 0.96 | 0.82, 1.12 |
|  | 15 | 0.95 | 0.80, 1.12 |
| **Infection (adjusted for †, ‡, §)** | 1 | 1.20 | 1.07, 1.33 |
|  | 3 | 1.08 | 0.98, 1.19 |
|  | 5 | 0.99 | 0.86, 1.14 |
|  | 7 | 0.94 | 0.79, 1.12 |
|  | 10 | 0.90 | 0.73, 1.11 |
|  | 13 | 0.88 | 0.70, 1.10 |
|  | 15 | 0.87 | 0.68, 1.10 |
| **Instability (adjusted for †, ‡, §)** | 1 | 1.01 | 0.87, 1.17 |
|  | 3 | 1.01 | 0.90, 1.12 |
|  | 5 | 1.01 | 0.89, 1.16 |
|  | 7 | 1.02 | 0.87, 1.19 |
|  | 10 | 1.02 | 0.85, 1.23 |
|  | 13 | 1.02 | 0.83, 1.26 |
|  | 15 | 1.03 | 0.82, 1.28 |
| **Pain (adjusted for †, ‡, §)** | 1 | 0.91 | 0.76, 1.09 |
|  | 3 | 1.04 | 0.90, 1.21 |
|  | 5 | 1.03 | 0.87, 1.21 |
|  | 7 | 0.98 | 0.76, 1.25 |
|  | 10 | 0.91 | 0.60, 1.37 |
|  | 13 | 0.87 | 0.51, 1.47 |
|  | 15 | 0.86 | 0.49, 1.51 |
| **Progression of OA (adjusted for †, ‡, §)** | 1 | 1.13 | 0.88, 1.45 |
|  | 3 | 1.40 | 1.17, 1.66 |
|  | 5 | 1.14 | 0.96, 1.37 |
|  | 7 | 0.95 | 0.74, 1.22 |
|  | 10 | 0.78 | 0.54, 1.13 |
|  | 13 | 0.69 | 0.44, 1.08 |
|  | 15 | 0.66 | 0.41, 1.06 |
| HR, hazard ratio; CI, confidence interval; OA, osteoarthritis; ASA, American Society of Anaesthesiologists grade. **^†^Patient factors:** age; sex; ASA; index of multiple deprivation decile. **^‡^Operation factors:** anaesthetic; approach; fixation; constraint; patellar resurfacing. **^§^Healthcare setting factors:** funding; year of operation. | | | |
