# Supplementary material for: Association between surgeon training grade and the risk of revision following total knee replacement: An analysis of National Joint Registry data
Source: PLoS Med. 2025 Aug 12;22(8):e1004685. doi: 10.1371/journal.pmed.1004685 (PMC12370202; doi:10.1371/journal.pmed.1004685)
Supplement: S1 Note — (DOCX) [file pmed.1004685.s009.docx]

**S1 Note – Considerations Influencing All-Cause Revision.**

Please note, the following supplementary discussion has been added in response to a comment raised by one of the reviewers during the peer-review process.

The primary outcome of this study was all-cause revision, defined by the NJR as “any procedure to add, remove, or modify one or more components of an implant construct for any reason.”

While the NJR robustly captures revision procedures across England and Wales, several external factors may influence whether and when a revision occurs. These include:

- **Follow-up consultation practices**: There is variability in post-operative follow-up across institutions and over time, particularly in how routine follow-up is structured beyond the first year. However, patients with concerning symptoms or functional decline are typically re-referred to orthopaedic services via primary care, and most revisions are captured regardless of formal follow-up arrangements.
- **Healthcare funding and cost considerations**: As the UK’s National Health Service (NHS) provides care free at the point of delivery, direct patient costs or insurance status do not influence revision decisions. This differs from other healthcare systems and reduces potential financial barriers to revision surgery.
- **Patient-reported outcomes and quality of life**: Although PROMs and functional scores may influence a clinician’s decision to revise, they are not routinely linked to NJR data. Therefore, we were not able to assess the association between PROMs and episodes of revision surgery in the context of surgical training.

In summary, while we acknowledge that individual clinical decisions around revision are complex and multifactorial, the use of all-cause revision within the NJR represents a consistently applied, objective, and widely accepted outcome measure in joint replacement research.
