# Supplementary material for: Association between surgeon training grade and the risk of revision following total knee replacement: An analysis of National Joint Registry data
Source: PLoS Med. 2025 Aug 12;22(8):e1004685. doi: 10.1371/journal.pmed.1004685 (PMC12370202; doi:10.1371/journal.pmed.1004685)
Supplement: Attachment — Submitted filename: Response.docx [file pmed.1004685.s013.docx]

Mr T J Fowler

Musculoskeletal Research Unit

Translational Health Sciences

Bristol Medical School

Southmead Hospital, Bristol

United Kingdom

**Prof. Dr. Till Bärnighausen**

Editor-in-Chief

PLOS Medicine

May 9th, 2025

Dear **Prof. Dr. Bärnighausen**,

**Reply to comments: “Association between surgeon training grade and the risk of revision following total knee replacement: an analysis of a National Joint Registry Data” (PMEDICINE-D-24-04032R1)**

Thank you for taking the time to review our study and for the detailed appraisal of our work. We would like to thank the individual reviewers for their time and constructive comments. I am pleased to attach our revised article, as well as our detailed response to each of the reviewers’ comments. We have attempted to address all comments and, where indicated, have edited the manuscript accordingly. We feel that the changes that we have made in response to reviewer’s comments have improved the quality of the manuscript as a whole.

Both reviewers and the editorial team have requested a number of additional analyses. We have endeavoured to incorporate these into this study where possible and where appropriate. This has not been possible in all cases, and we explain the reasons for this in detail. Please note, the analyses included in this paper are consistent with those performed in our previous publications on this subject, including our recent related publication in PLoS Medicine.

<https://doi.org/10.1371/journal.pmed.1004445>

Yours sincerely,

Mr Tim Fowler

BM BCh (Oxon) MA MD FRCS

Academic Clinical Fellow & Orthopaedic Registrar

Musculoskeletal Research Unit, Bristol Medical School, Southmead Hospital, Bristol, UK

Email: t.j.fowler@bristol.ac.uk; Phone: +44 (0)117 414 7865

| **Reply to comments: “Association between surgeon training grade and the risk of revision following total knee replacement: an analysis of a National Joint Registry Data” (PMEDICINE-D-24-04032R2)** | | |
| --- | --- | --- |
|  | | |
| **Reviewer & Comment number** | **Comment** | **Response** |
| **Reviewer 1: Comment 1** | *This is an interesting registry based analysis of outcome of TKR compared to the level of surgical training concluding that there was little difference in revision rate between unsupervised and supervised trainees compared to consultant surgeons.*  *This data supplements previous studies showing similar results but has the value of being a very large data base. The authors looked at reasons for revision and included this as one of the endpoints for comparison, which had not been studied before.*  *They concluded that there was a small increase in revision rate for trainees, in particular they highlighted patellar resurfacing as an important difference between trainees and consultants and recommended all knees be resurfaced (based on their previous publication). Patellar resurfacing is a topic of debate, in particular resurfacing as a secondary procedure, which has often been criticized as not improving patient outcomes or relieving pain.*  *The authors need to restrain their conclusions and offer a more reasoned approach to the small difference they have found as they offer no further data on outcomes of revision surgery in these cases i.e. did the revision actually improve the outcome. The threshold to revise TKR performed by a trainee may be lower and at least needs to be acknowledged by the authors.* | We thank the reviewer for the thoughtful appraisal and constructive feedback.  We have been careful throughout the manuscript to avoid overstating our conclusions. Our findings are presented as associations rather than causal inferences, and we clearly acknowledge the limitations inherent in observational registry data. We have taken care to use balanced language throughout the manuscript when interpreting the findings.  Regarding patellar resurfacing, we do not recommend routine resurfacing in all cases. In the discussion (paragraph 4), we refer to the UK NICE guidelines, which recommend that patellar resurfacing be offered to all patients undergoing TKR. Based on this guidance, we suggest that trainers should consider resurfacing, particularly when a trainee is performing the procedure. We conclude the paragraph with the sentence: *“We propose that trainers should consider patellar resurfacing, particularly when the trainee performs the TKR.”* This is intended to reflect current evidence-based recommendations and the importance of structured intraoperative decision-making during training.  Later in the discussion, we also state: *“TKRs may be susceptible to an increased risk of early revision for aseptic loosening, infection, and progression of OA. We recommend that consultants and trainees should recognise and take appropriate measures to reduce the risk of failure from these indications. Trainees should ideally be supervised by a scrubbed consultant when performing TKR, particularly when junior and at critical stages of the procedure such as implant selection, balancing, fixation, and deciding whether or not to resurface the patella.”*  We agree with the reviewer that the outcomes of revision surgery are not captured within the NJR, and as such, we cannot comment on whether these revisions improved patient symptoms or satisfaction. This is covered in our discussion of the limitations:  *“Implant survival is an important objective measure of success. However, we did not consider other metrics that may be relevant when evaluating the success of a TKR, such as patient-reported outcome measures (PROMS), or postoperative complications other than failure, as they are not currently reported by the NJR [1].”*  We also recognise the possibility that trainee-performed TKRs may be revised at a lower threshold, though we have no data to confirm this. We have also addressed this in the revised limitations section:  *“We also acknowledge the possibility that primary TKRs performed by trainees may be subject to a lower threshold for revision, particularly in the early postoperative period. This could reflect differing decision-making thresholds among supervising teams, though this could not be assessed within the available data.”* |
| **Reviewer 2: Comment 1** | In the Patients and data sources subsection, it is stated that inclusions were for indication of OA only. Was there any specific reason as to why other indications for TKR were not considered? | We are very grateful for the time you have taken to review our work and for your constructive comments. We hope to have addressed each of these in a satisfactory manner.  TKRs performed for other indications (e.g. inflammatory arthropathy, RA, malignancy, SONK, AVN, trauma) would generally be considered more complex, and thus may be less likely to be performed by a trainee. Furthermore, TKRs performed for these indications are susceptible to a higher risk of revision.  The decision to only include cases performed for an indication of OA was made in the interest of relative standardisation of procedural complexity. An alternative approach would have been to adjust the analysis for the indication. However, it is common practice in studies based on NJR data to only include cases performed for OA. Adjusting the analysis for indication would add unnecessary statistical complexity, when the vast majority of primary TKRs are performed for OA.  We have now added the following sentence to the ‘patient and data sources’ section of the methods:  *“The analysis was restricted to cases performed for an indication of OA to facilitate relative standardisation of procedural complexity and limit confounding by indication”*  Furthermore, we have added more discussion on this matter to the limitations subsection of the discussion:  *“We have attempted to account for measurable variations in case complexity between the groups by adjusting for a comprehensive range of confounding variables and by restricting the analysis to cases performed for OA, which may help reduce confounding by indication…”* |
| **Reviewer 2: Comment 2** | In the Data access and processing subsection, the inclusion process is described and illustrated in Figure 1 and S1/S2 Figure. However, "complete survival data" as detailed in S2 Figure does not appear to describe the completeness of temporal data. It might thus be clarified as to the requirements for follow-up data (i.e. if a patient does not have any visits recorded after the original surgery, is he considered as not requiring any revisions?) | We appreciate your comment. With regards to the completeness of survival data/follow-up data, we would like to draw your attention to the NJR data linkage process and data quality audits. Inclusion of all major joint replacements performed in the UK in the NJR has been mandatory since 2011. This process is discussed in detail within the NJR Annual Report, which we have cited throughout the paper.  The NJR data quality process has been published in the following article: <https://doi.org/10.1302/2633-1462.39.BJO-2022-0051.R1>. This describes the rigorous processes undertaken by the NJR to ensure that data for primary and revision procedures are complete. NJR data are over 95% complete at baseline, increasing to over 97% nationally after the audit process.  Revision data: The NJR links records of primary joint replacements to episodes of revision.  Mortality data: NJR data are annually linked to data from other healthcare systems using patient identifiers, principally a patient’s NHS number. These other datasets include the Hospital Episodes Statistics (HES) service, and Civil Registration data (all provided by NHS Digital), and the Patient Episode Database Wales (PEDW) (provided by NHS Wales Informatics Service).  NB: These processes of data linkage and data quality audit were carried out by the NJR (not the authors) prior to us obtaining access to the dataset, which we clarify in the manuscript. The base dataset used in our study is the same as that used in the NJR 17^th^ Annual Report, which we cite. The base dataset only consists of records with complete survival data.  In response to your comment, we have now edited this section to read as follows, and included a citation to the NJR data quality process:  *“The base dataset used in the current study is based on the same cut of NJR data that is used in the 17^th^ Annual Report [2]. NJR data are annually linked to other healthcare system datasets, including the Hospital Episodes Statistics (HES) service, and Civil Registration Authority data, using unique patient identifiers. This linkage, which was carried out by the NJR prior to us obtaining the dataset, is approved by the Health Research Authority under Section 251 of the NHS act 2006 [2]. NJR data completeness is routinely monitored using a published data quality audit process.”* |
| **Reviewer 2: Comment 3** | In the Exposures subsection, it is stated that the secondary exposure was whether or not trainees were directly supervised by a scrubbed consultant during the procedure. It appears likely that there may be a supervision bias by trainee grade (i.e. F1 less likely to be unsupervised, compared to fellows). Moreover, the relevant figures (Figures 3 to 5) do not appear to compare revision hazard ratio by supervision status. This might be addressed. | Many thanks for your recommendation. Please see Figure 4 (and the associated legend), which illustrates the risk of all-cause revision of TKRs according to the level of supervision of trainees (exposure B). Figure 4 is a HR plot for a fully adjusted flexible parametric survival model (Model 4). It represents TKRs performed by trainees supervised by a scrubbed consultant (baseline) compared to TKRs performed by trainees not supervised by a scrubbed consultant (exposure B).  We discuss Figure 4 in the third paragraph of the “All-cause revision: flexible parametric survival modelling (FPM)” subsection of the results, where we state:  *“Further analysis was performed to compare the risk of revision of TKRs performed by trainees who were supervised by a scrubbed consultant to TKRs performed by trainees who were not supervised by a scrubbed consultant (exposure B). There was no evidence of an association between the level of scrubbed consultant supervision of trainee-performed TKRs and the risk of all-cause revision (Figure 4).”*  We acknowledge that we have only performed this analysis for trainees overall, rather than for F1-ST2; ST3-8 and fellows.  As we already discuss in the *“All-cause revision: flexible parametric survival modelling (FPM)”* subsection of the results, only a very small proportion of trainee procedures were performed by F1-ST2 trainees (1.1%) and fellows (3.4%). ST3-8 trainees performed 95.4% of all trainee TKRs. As such, the number of F1-ST2 and fellow cases/revision events is too low to conduct the analysis using the model we have developed. The model simply does not work with such low numbers (we have even tried to simply the model, but to no avail).  We can however present the results for ST3-8 trainees according to the level of supervision (as 95.4% of the trainee cases are performed by trainees). We have included this in the paper, with a new Figure 6 (the old Fig 6 is nor Fig 7). We reference this in the text as follows:  *“Furthermore, there was no evidence of an association between the level of scrubbed consultant supervision of TKRs performed by ST3-ST8 surgeons and the risk of all-cause revision (Figure 6). There were insufficient cases performed by F1-ST2 surgeons and fellows to repeat this analysis for these groups.”* |
| **Reviewer 2: Comment 4** | i) Additionally, the choice of case assignment to trainees might be considered - would there be bias regarding the expected complexity of the case (i.e. easier/more routine cases would be assigned to trainees)? ii) Related to this, Table 1 suggests that the proportion of cases handled by consultants versus trainees is significantly higher in 2012-2019 as compared to 2003-2011. This might be briefly discussed. | i) This is an excellent point, which is also touched upon by another reviewer (Reviewer 3: Comments 8+9). In response to your comment we have now revised the opening paragraph in the ‘limitations’ subsection of the discussion to read as follows:  *“Despite the strengths of this study, it has limitations. The data are observational, and patients were not randomly allocated to intervention groups; therefore, causation cannot be inferred. It is likely that more complex cases are preferentially performed by consultants. We have attempted to account for measurable differences in case complexity between the groups by adjusting for a comprehensive range of confounding variables and by restricting the analysis to cases performed for OA, which may help reduce confounding by indication. Furthermore, additional sensitivity analyses were conducted, including: i) adjustment for BMI, and ii) restriction to NHS-funded procedures in NHS hospitals, excluding procedures performed in private hospitals where trainees in England and Wales do not routinely operate. However, we acknowledge that residual confounding remains likely, and to our knowledge, no further adjustments are possible for factors influencing case allocation to trainees. Nonetheless, our findings suggest that the current system for allocating cases to trainees in England and Wales is safe.”*  ii) Another excellent point. Please see our response to Reviewer 3: Comment 3 for a more detailed discussion. We have now added the following to the results section:  “*Trainees have performed a lower proportion of cases since 2012 (9.1% since 2012 vs. 12.0% pre-2012) and a higher proportion of trainee procedures have been supervised by a scrubbed consultant since 2012 (64.0% since 2012 vs. 36.0% pre-2012).”* |
| **Reviewer 2: Comment 5** | In the Outcomes of interest subsection, all-cause revision was stated as the primary outcome measure. The typical process of diagnosis/recommendation for such revision could be briefly discussed. | Thank you for your comment. We already cite the NJR definition for the diagnosis of all-cause revision. This is:  *“…any procedure to add, remove, or modify one or more components of an implant construct for any reason.”*  We feel that any discussion of revision surgery thresholds, or specific diagnostic criteria for different indications for revision, would be out of place in this subsection and might be confusing for the reader. We feel that it is important to keep this subsection clear and concise. |
| **Reviewer 2: Comment 6** | Figure 5 suggests higher revision for fellows, >4 years since primary procedure. This might be commented on - does this indicate a general delay in revisions compared to F1-ST8, or an overall increased rate of revisions over 16 years? | The lower 95% CI for fellow-performed TKRs (noted in Fig. 5), briefly does not overlap the baseline of 1 at the 6-year timepoint. It overlaps the baseline at all other intervals between 0-16 years, indicating no association between fellow-performed TKR and all-cause revision (at all but possibly year 6).  Please note, we highlight in the text that only a very small proportion of trainee cases were performed by fellows (3.4%) and F1-ST2 doctors (1.1%). Thus, this is very unlikely to be of any significance in the context of trainee-performed cases as a whole. |
| **Reviewer 2: Comment 7** | For Figure 5, it might be considered to standardize the scale of the y-axis for all charts (possibly increasing the height if required), for easier comparison; the corresponding chart for consultants could also be included. | Thank you for your recommendation. We have revised Figure 5 to standardise the scale of the y-axis. Please note that the baseline of 1 on each plot represents consultant-performed TKR, thus a separate consultant plot is not required. Each plot represents comparison to consultant-performed TKR. |
| **Reviewer 2: Comment 8** | In Figures 3 to 6, the 95% CIs for many of the charts appears similar at 0 years and 16 years, where it would be expected to be wider at 16 years due to a relative lack of data (from about 2003 only), as compared to at 0 years (where all data from 2003-2019 is relevant; this is reflected as expected in the Kaplan-Meier plots in Figure 2). These estimates might be confirmed. | We acknowledge your point regarding the breadth of CIs with time. We can confirm that the code for each plot has been rechecked and confirmed. Please note that the KM plots and FPM Hazard Ratio plots are distinct forms of analysis, as discussed in the Statistical analysis subsection.  The 1-KM plots display the cumulative probability of failure/revision. Hazard ratio plots display the HR/risk of revision at a specific instant in the time of follow-up. These are calculated using FPM which accounts for time-varying effects in the data. This accounts for the tendency for more consistent CIs throughout follow-up.  Lambert and Royston’s papers on FPM are cited in the manuscript. |
| **Reviewer 2: Comment 9** | In the Statistical analysis subsection, it is stated that records were censored at the end of 2019 or date of death, whichever is earliest. It might be clarified as to whether other factors (e.g. emigration) were considered. | We can confirm that cases were not censored on the date of emigration.  It is possible that episodes of revision surgery may not be captured in NJR data if patients have emigrated and subsequently have revision surgery overseas. However, the number of TKRs revised overseas is likely to be very low and unlikely to be influenced by the grade of the surgeon who performed the primary procedure.  With regards to death overseas: NJR data are linked to Civil Registration data (i.e. mortality data. See discussion in Comment 2). UK mortality records capture overseas mortality when cases of overseas mortality are reported through this British consulate in the country of death. Again, this is unlikely to be influenced by the grade of the surgeon who performed the primary procedure.  This is an interesting topic, but in the interest of keeping the paper concise, we do not feel that discussion of this topic within our paper would add value. |
| **Reviewer 2: Comment 10** | In the Statistical analysis subsection, further details about FPM modelling were stated to be described in S2 Appendix. AIC & BIC details across various parameters such as degrees of freedom and knots might be included, if possible. | According to your recommendation, we have now included AIC and BIC details for degrees of freedom (DF) and degrees of freedom for time-varying effects (DFTVE) within S2 Appendix. |
| **Reviewer 3:**  **Comment 1** | This is an interesting subject, and the authors have attempted to answer the question of whether trainee performed TKR does as well as those performed by consultants. The methodology uses the NJR dataset, the limitations of which are well known and to a certain degree acknowledged in the paper. The aim of this paper should be to either change practice or confirm that current practice is acceptable. However, the analysis as reported is too simplistic to achieve either of these aims. | We are very grateful for the time you have taken to review our work and for your constructive comments. |
| **Reviewer 3:**  **Comment 2** | The weaknesses are considerable; the NJR dataset is non-verified for quality of what is input, there is no adequate case mix identification between groups, BMI is omitted due to missing values early in the NJR, revision for primary joint replacement may be a weak marker of "success" as opposed to PROMs or patient satisfaction measures, the disparity between consultant and trainee groups and the mix of settings (NHS v independent sector).  However, I think this is an important subject and it should be possible to obtain a more useful analysis. | We agree that this is an important subject and feel that the paper is improved following the incorporation of some of the reviewer’s recommendations.  We acknowledge the limitations of this study, which primarily relate to the observational nature of registry data. We have discussed key limitations of the study in the revised discussion section.  For further information on the measures taken to verify the quality of NJR data please see the following, which we have now referenced in the paper: <https://doi.org/10.1302/2633-1462.39.BJO-2022-0051.R1>.  You have recommended a number of additional analyses. We have endeavoured to incorporate these into this study where possible and where appropriate. In some cases, it has not been possible to include additional analyses. This is due to limitations of NJR data and due to the fact that we only have permission from the NJR to conduct analsyses on this specific subject (surgeon training grade). We address each of your comments detail below.  Please note, the analyses included in this paper are consistent with those performed in our previous publications on this subject. Including our recent related publication in PLoS Medicine [3, 4]. |
| **Reviewer 3:**  **Comment 3** | I wonder whether the authors would consider a more in depth analysis? Even with only approximately 10% of TKRs being performed by trainees the number available to analyse are considerable.  My suggestions would be:  Training has altered considerably since 2003. Trainees now perform considerably fewer procedures overall. Is there a difference in revision rate for trainee performed TKR over time? | We agree that the nature of surgical training has evolved significantly over the past two decades and we have considered this in our models.  The ‘year of operation’ is summarised in Table 1 and adjusted for in our statisitical models. Please see the following in the statistical analysis subsection: *“Model 4 was further adjusted for healthcare setting factors (funding source, and year of operation).”*  We break down the cases according to whether they were performed pre- or post 2012. The rationale for this date being used, is that it represents the point at which it became mandatory for all TKRs performed in England and Wales to be reported to the NJR. One might argue that manadatory NJR data entry may have reduced training opportunities.  Table 1 confirms that a lower proportion of cases have been performed by trainees since 2012. 2003-2011: 41,112/342,217 = 12% vs. 2012-2019: 55,432/610,864 = 9.1%. Table 1 also indicates that a higher proportion of trainee procedures have been supervised by a scrubbed consultant since 2012.  As stated, the ‘year of operation’ is already reported and accounted for in our models. Thus, we do not feel it is indicated to add an additional analysis in this case. However, in repsonse to your comment, we have now added the following to the results section:  “*Trainees have performed a lower proportion of cases since 2012 (9.1% since 2012 vs. 12.0% pre-2012) and a higher proportion of trainee procedures have been supervised by a scrubbed consultant since 2012 (64.0% since 2012 vs. 36.0% pre-2012).”* |
| **Reviewer 3:**  **Comment 4** | Is there a difference in revision rate between trainees who have only TKRs registered as a trainee versus those trainees who have subsequent TKRs registered as a consultant? | We thank the reviewer for this insightful suggestion. We agree that comparing revision rates between trainees who later go on to perform TKRs as consultants versus those who do not would be a valuable and informative analysis. Unfortunately, this is not feasible with current NJR data. While some trainee-performed cases are recorded using a unique Surgeon ID that would enable longitudinal tracking of an individual trainee’s practice, in many instances, trainee cases are instead recorded under the supervising consultant’s ID. This inconsistency in how trainee procedures are attributed means it is not possible to reliably identify and follow individual trainees through to their later consultant practice. Therefore, we are unable to perform the suggested analysis. Trainees are only given a unique surgeon ID number when they apply for one, otherwise their cases are recorded using the ID of the supervising consultant. This excplanation is applicable to a number of the other anlayses that you have recommended, which would have required us to attribute cases to individual trainees. We do not feel that this warrants discussion in the paper as these analyses were not planned as part of our study protocol. However, we would be happy to mention this in the discussion if the editorial team deem this necessary. |
| **Reviewer 3:**  **Comment 5** | Is there a difference in trainee revision rate as their numbers increase? e.g. does revision rate fall as experience increases? Is there a number of TKRs performed where the revision rate plateaus? Can you define the ranges of a learning curve? | Another good suggestion, but for reasons explained above, this is not feasible when looking at cases done by trainees. Furthermore, please note that ‘surgeon grade’, which is the primary subject of this paper, and ‘learning curve’ are separate subjects. We do not have the necessary permissions from the NJR to perform additional analsyses on the ‘learning curve’ of TKRs.  Some background on this:  The term ‘learning curve’ is generally used to describe a surgeon’s rate of skills acquisition during an early period of practice, or the minimum number of cases required for a surgeon to become “competent” in a specific procedure. The term has been widely used in the orthopaedic literature, including in the context of training in hip and knee replacement [5-8].  The term ‘surgeon grade’ is a commonly used measure of surgical experience, which is used to define a surgeon’s designated level of training (e.g. trainee or consultant).  In the existing orthopaedic literature, ‘surgeon grade’ has been used as a binary variable to describe whether a procedure has been performed by a trainee or a consultant [9-13]. It is also established practice for trainee-performed procedures to be subcategorised according to whether or not a trainee was supervised by a consultant [14, 15]. |
| **Reviewer 3:**  **Comment 6** | Is the revision rate of unsupervised trainee TKRs affected by the number of supervised TKRs the trainee performed? | As above: Please see our detailed response to Comment 4. |
| **Reviewer 3:**  **Comment 7** | In terms of comparison to consultant performance, limiting the consultant group to NHS procedures in NHS hospitals would limit the confounders as you are then comparing within the same population (I assume just about all the trainee procedures are in NHS hospitals). | We thank the reviewer for this helpful suggestion. We agree that restricting the analsysis to NHS-funded procedures procedures in NHS hospitals improves the comparability of the cohorts by reducing healthcare setting-related confounding. We have now added this additional analysis to the manuscript and updated the relevant results and interpretation accordingly. Please see revised Figure 3, which now also includes the results of this sensitivity analysis. |
| **Reviewer 3:**  **Comment 8** | Reconsider not omitting BMI. I appreciate that there are many missing entries in the earlier years, but it is a good indicator of difficulty and there is an increased risk of infection in high BMI and infection is one of the main reasons for revision in the trainee group. | We thank the reviewer for this valuable suggestion. We agree that BMI is an important clinical variable, particularly in relation to infection risk. Although BMI is missing in a substantial proportion of cases, particularly in the earlier years of the registry, we have now included BMI in a sensitivity analysis limited to cases with complete BMI data. This analysis is described in the revised Methods and Results sections, and the impact of BMI is discussed in the context of our findings. Please see revised Figure 3, which now also includes the results of this sensitivity analysis. |
| **Reviewer 3:**  **Comment 9** | The FFD and ROM questions on the form allow some quantification of difficulty of the procedure. Does a FFD affect revision rate? It may then be possible to either confirm current practice is safe, but it might also provide some insight into how the chance of a poor outcome may be minimised and training optimised. | This is an interesting point, which highlights the issue of residual confounding relating to case complexity and case-mix selection.  We would have liked to use such variables in our analysis in order to further account for variations in case complexity between the groups. Unfortunately, the FFD and ROM data were not included in the base dataset provided by the NJR. We do not have access to this data, and it is not included in the base dataset used for the NJR Annual Report analysis.  FFD and ROM were not included on early versions of the NJR Minimum Data Set (MDS) form, and these data are incomplete on a large proportion of forms. Furthermore, the ROM and FFD assessment is a subjective measure, unlike categorical variables, such as training grade. We are not aware of these measures previously being used in statistical models in NJR papers.  We have revised the first paragraph of our limitations section to address the issue of residual confounding. We can confirm that, to our knowledge, there are no further steps to take to adjust for factors that might have influenced the allocation of cases to trainees. This edited paragraph reads as follows:  *“Despite the strengths of this study, it has limitations. The data are observational, and patients were not randomly allocated to intervention groups; therefore, causation cannot be inferred. It is likely that more complex cases are preferentially performed by consultants. We have attempted to account for measurable differences in case complexity between the groups by adjusting for a comprehensive range of confounding variables and by restricting the analysis to cases performed for OA, which may help reduce confounding by indication. Furthermore, additional sensitivity analyses were conducted, including: i) adjustment for BMI, and ii) restriction to NHS-funded procedures in NHS hospitals, excluding procedures performed in private hospitals where trainees in England and Wales do not routinely operate****. However, we acknowledge that residual confounding remains likely, and to our knowledge, no further adjustments are possible for factors influencing case allocation to trainees****. Nonetheless, our findings suggest that the current system for allocating cases to trainees in England and Wales is safe*.” |
| **General Editorial Request: 1** | *Please upload any figures associated with your paper as individual TIF or EPS files with 300dpi resolution at resubmission; please read our figure guidelines for more information on our requirements:* [*http://journals.plos.org/plosmedicine/s/figures*](http://journals.plos.org/plosmedicine/s/figures)*. While revising your submission, please upload your figure files to the PACE digital diagnostic tool, https://pacev2.apexcovantage.com/. PACE helps ensure that figures meet PLOS requirements. To use PACE, you must first register as a user. Then, login and navigate to the UPLOAD tab, where you will find detailed instructions on how to use the tool. If you encounter any issues or have any questions when using PACE, please email us at PLOSMedicine@plos.org.* | This has been addressed. |
| **General Editorial Request: 2** | *Please ensure that the paper adheres to the PLOS Data Availability Policy*  *(see* [*http://journals.plos.org/plosmedicine/s/data-availability)*](http://journals.plos.org/plosmedicine/s/data-availability))*, which requires that all data underlying the study's findings be provided in a repository or as Supporting Information. For data residing with a third party, authors are required to provide instructions with contact information (web or email address) for obtaining the data. Please note that a study author cannot be the contact person for the data. PLOS journals do not allow statements supported by "data not shown" or "unpublished results." For such statements, authors must provide supporting data or cite public sources that include it.* | Please see our data availability statement, which reads as follows:  “The data used in the study are available from The National Joint Registry (NJR) (https://www.njrcentre.org.uk). Restrictions apply to the availability of these data, which were used under license for the current study, and are therefore not publicly available. Data access applications can be made to the NJR Research Committee. With NJR permission in place, the data underlying the presented results will be available to access via the NJR data access network. The authors of this manuscript are not the data owner and do not have permission to share the data.” |
| **General Editorial Request: 3** | *We expect all researchers with submissions to PLOS in which author-generated code underpins the findings in the manuscript to make all author-generated code available without restrictions upon publication of the work. In cases where code is central to the manuscript, we may require the code to be made available as a condition of publication. Authors are responsible for ensuring that the code is reusable and well documented. Please make any custom code available, either as part of your data deposition or as a supplementary file. Please add a sentence to your data availability statement regarding any code used in the study, e.g. "The code used in the analysis is available from Github [URL] and archived in Zenodo [DOI link]" Please review our guidelines at* [*https://journals.plos.org/plosmedicine/s/materials-software-and-code-sharing*](https://journals.plos.org/plosmedicine/s/materials-software-and-code-sharing) *and ensure that your code is shared in a way that follows best practice and facilitates reproducibility and reuse. Because Github depositions can be readily changed or deleted, we encourage you to make a permanent DOI'd copy (e.g. in Zenodo) and provide the URL.* | We are happy to make author-generated code for this paper available on publication. This code is for the statistical analysis of NJR data using Stata. |
| **General Editorial Request: 4** | *Abstract: Please structure your abstract using the PLOS Medicine headings (Background, Methods and Findings, Conclusions). Please combine the Methods and Findings sections into one section.* | This has been addressed. |
| **General Editorial Request: 5** | *At this stage, we ask that you include a short, non-technical Author Summary of your research to make findings accessible to a wide audience that includes both scientists and non-scientists. The Author Summary should immediately follow the Abstract in your revised manuscript. This text is subject to editorial change and should be distinct from the scientific abstract. Ideally each sub-heading should contain 2-3 single sentence, concise bullet points containing the most salient points from your study. In the final bullet point of 'What Do These Findings Mean?', please include the main limitations of the study in non-technical language. Please see our author guidelines for more information:* [*https://journals.plos.org/plosmedicine/s/revising-your-manuscript#loc-author-summary*](https://journals.plos.org/plosmedicine/s/revising-your-manuscript#loc-author-summary)*.* | An Author Summary has now been included. |
| **General Editorial Request: 6** | *Please express the main results with 95% CIs as well as p values. When reporting p values please report as p<0.001 and where higher as the exact p value p=0.002, for example. Throughout, suggest reporting statistical information as follows to improve clarity for the reader "22% (95% CI [13%,28%]; p</=)". Please be sure to define all numerical values at first use.* | We have reviewed the manuscript and made changes where necessary. This has now been addressed. |
| **General Editorial Request: 7** | *Please include page numbers and line numbers in the manuscript file. Use continuous line numbers (do not restart the numbering on each page).* | Page and line numbers have now been included. |
| **General Editorial Request: 8** | *Please cite the reference numbers in square brackets. Citations should precede punctuation.* | This has been addressed. |
| **General Editorial Request: 9** | *Please provide titles and legends for all figures and tables (including those in Supporting Information files).* | This has been addressed. |
| **General Editorial Request: 10** | *Please define all abbreviations used in each figure/table (including those in Supporting Information files).* | This has been addressed. |
| **General Editorial Request: 11** | *Please consider avoiding the use of red and green in order to make your figure more accessible to those with color blindness.* | This has been addressed. |
| **General Editorial Request: 12** | *Please note that supplementary material will be posted as supplied by the authors. Therefore, please amend it according to the relevant comments outlined here.* | This has been addressed. |
| **General Editorial Request: 13** | *Please cite your Supporting Information as outlined here:* [*https://journals.plos.org/plosmedicine/s/supporting-information*](https://journals.plos.org/plosmedicine/s/supporting-information) | This has been addressed. |
| **General Editorial Request: 14** | *PLOS uses the numbered citation (citation-sequence) method and first six authors, et al.* | This has been addressed. |
| **General Editorial Request: 15** | *Please ensure that journal name abbreviations match those found in the National Center for Biotechnology Information (NCBI) databases (*[*http://www.ncbi.nlm.nih.gov/nlmcatalog/journals*](http://www.ncbi.nlm.nih.gov/nlmcatalog/journals)*), and are appropriately formatted and capitalised.* | This has been addressed. |
| **General Editorial Request: 16** | *Where website addresses are cited, please include the complete URL and specify the date of access (e.g. [accessed: 12/06/2023]).* | This has been addressed. |
| **General Editorial Request: 17** | *Abstract: Please include the study design, population and setting, number of participants, years during which the study took place (enrolment and follow up), length of follow up, and main outcome measures.* | This has been addressed. |
| **General Editorial Request: 18** | *Please ensure that the study is reported according to the STROBE (or appropriate STOBE extension) guideline (available from:* [*https://www.equator-network.org/reporting-guidelines/strobe)*](https://www.equator-network.org/reporting-guidelines/strobe)) *and include the completed STROBE (or STROBE extension) checklist as Supporting Information. Please add the following statement, or similar, to the Methods: "This study is reported as per the Strengthening the Reporting of Observational Studies in Epidemiology (STROBE) guideline (S1 Checklist)." When completing the checklist, please use section and paragraph numbers, rather than page numbers.* | This study is reported as per the Reporting of studies Conducted using Observational Routinely-collected health Data (RECORD) Statement (S1 Checklist) [16].  This is an extension of STROBE, which is designed for studies conducted using routinely-collected health data. The RECORD checklist is endorsed by PLoS Medicine and specific to this study type. |
| **General Editorial Request: 19** | *For all observational studies, in the manuscript text, please indicate: (1) the specific hypotheses you intended to test, (2) the analytical methods by which you planned to test them, (3) the analyses you actually performed, and (4) when reported analyses differ from those that were planned, transparent explanations for differences that affect the reliability of the study's results. If a reported analysis was performed based on an interesting but unanticipated pattern in the data, please be clear that the analysis was data driven.* | We have fulfilled this within the manuscript. |
| **General Editorial Request: 20** | *Please state in the Methods section whether the study had a prospective protocol or analysis plan. If a prospective analysis plan (from your funding proposal, IRB or other ethics committee submission, study protocol, or other planning document written before analyzing the data) was used in designing the study, please include the relevant document(s) with your revised manuscript as a Supporting Information file to be published alongside your study and cite it in the Methods section. A legend for this file should be included at the end of your manuscript. If no such document exists, please make sure that the Methods section transparently describes when analyses were planned, and when/why any data-driven changes to analyses took place. Changes in the analysis, including those made in response to peer review comments, should be identified as such in the Methods section of the paper, with rationale.* | The study protocol was planned in detail prior to commencing the study, including defining the study population, exposures, outcomes of interest and statistical methods. The main analyses were planned prior to commencing the study and these are documented throughout the methods section. However, data-driven changes to the analysis took place and these are summarised in detail in S2 Appendix, which justifies the model selection and construction.  The study protocol was not published prior to commencing the study. Thus, no such document exists. We have addressed this in the second paragraph of the methods section, which reads as follows:  *“****Analysis plan***  *The study protocol was designed prior to commencing the study, including defining the study population, exposures, outcomes of interest and statistical methods. The main analyses were planned prior to commencing the study and these are documented throughout this methods section. However, data-driven changes to the analysis took place and these are summarised in detail in S2 Appendix, which justifies the model selection and construction.”* |

**REFERENCES**

1.	Wylde V, Blom AW. The failure of survivorship. J Bone Joint Surg Br. 2011;93(5):569-70.

2.	NJR: 17th Annual Report 2020 [cited 2025 04/05/2025]. Available from: <https://reports.njrcentre.org.uk/Portals/0/PDFdownloads/NJR%2017th%20Annual%20Report%202020.pdf>.

3.	Fowler TJ, Aquilina AL, Reed MR, Blom AW, Sayers A, Whitehouse MR. The association between surgeon grade and risk of revision following total hip arthroplasty : an analysis of National Joint Registry data. Bone Joint J. 2022;104-B(3):341-51.

4.	Fowler TJ, Howells NR, Blom AW, Sayers A, Whitehouse MR. Association between surgeon training grade and the risk of revision following unicompartmental knee replacement: An analysis of National Joint Registry data. PLoS Med. 2024;21(9):e1004445.

5.	Nzeako O, Back D. Learning Curves in Arthroplasty in Orthopedic Trainees. J Surg Educ. 2016;73(4):689-93.

6.	McCulloch RA, Howgate D, Gibbs VN, Palmer A, Taylor AH, Kendrick B. Assessing the performance and learning curve of orthopaedic surgical trainees in primary total hip arthroplasty. Ann R Coll Surg Engl. 2021;103(7):514-9.

7.	de Steiger RN, Lorimer M, Solomon M. What is the learning curve for the anterior approach for total hip arthroplasty? Clin Orthop Relat Res. 2015;473(12):3860-6.

8.	Alvand A, Wilson HA, Sabah SA, Middleton R, Bottomley N, Jackson WFM, et al. New instrumentation system for cementless mobile-bearing unicompartmental knee arthroplasty improves surgical performance particularly for trainees. Knee. 2021;31:46-53.

9.	Bottomley N, Jones LD, Rout R, Alvand A, Rombach I, Evans T, et al. A survival analysis of 1084 knees of the Oxford unicompartmental knee arthroplasty: a comparison between consultant and trainee surgeons. Bone Joint J. 2016;98-B(10 Supple B):22-7.

10.	Palan J, Gulati A, Andrew JG, Murray DW, Beard DJ, group Es. The trainer, the trainee and the surgeons' assistant: clinical outcomes following total hip replacement. J Bone Joint Surg Br. 2009;91(7):928-34.

11.	Faulkner A, Reidy MJ, Davies PSE, Wang W, Buddhadev N, Johnston L, et al. Balancing training and outcomes in total knee replacement: A ten-year review. Surgeon. 2018;16(4):227-31.

12.	Jain S, Magra M, Dube B, Veysi VT, Whitwell GS, Aderinto JB, et al. Reverse hybrid total hip arthroplasty. Bone Joint J. 2018;100-B(8):1010-7.

13.	Reidy MJ, Faulkner A, Shitole B, Clift B. Do trainee surgeons have an adverse effect on the outcome after total hip arthroplasty?: a ten-year review. Bone Joint J. 2016;98-B(3):301-6.

14.	Inglis T, Dalzell K, Hooper G, Rothwell A, Frampton C. Does orthopedic training compromise the outcome in total hip arthroplasty? J Surg Educ. 2013;70(1):76-80.

15.	Storey R, Frampton C, Kieser D, Ailabouni R, Hooper G. Does Orthopaedic Training Compromise the Outcome in Knee Joint Arthroplasty? J Surg Educ. 2018;75(5):1292-8.

16.	Benchimol EI, Smeeth L, Guttmann A, Harron K, Moher D, Petersen I, et al. The REporting of studies Conducted using Observational Routinely-collected health Data (RECORD) statement. PLoS Med. 2015;12(10):e1001885.
