# Supplementary material for: Association between surgeon training grade and the risk of revision following total knee replacement: An analysis of National Joint Registry data
Source: PLoS Med. 2025 Aug 12;22(8):e1004685. doi: 10.1371/journal.pmed.1004685 (PMC12370202; doi:10.1371/journal.pmed.1004685)
Supplement: Attachment — Submitted filename: Response_auresp_3.docx [file pmed.1004685.s014.docx]

Mr T J Fowler

Musculoskeletal Research Unit

Translational Health Sciences

Bristol Medical School

Southmead Hospital, Bristol

United Kingdom

**Prof. Dr. Till Bärnighausen**

Editor-in-Chief

PLOS Medicine

June 30th, 2025

Dear **Prof. Dr. Bärnighausen**,

**Reply to comments: “Association between surgeon training grade and the risk of revision following total knee replacement: an analysis of a National Joint Registry Data” (PMEDICINE-D-24-04032R3)**

Thank you again for taking the time to review our paper and for the detailed appraisal of our work. Once again, we would like to thank the editorial team for their time in re-reviewing our work. I am pleased to attach our re-revised article, as well as our response to each of the editorial comments. We have addressed all of the comments and have edited the manuscript accordingly. We hope that you find these changes satisfactory.

Yours sincerely,

Mr Tim Fowler

BM BCh (Oxon) MA MD FRCS

Academic Clinical Fellow & Orthopaedic Registrar

Musculoskeletal Research Unit, Bristol Medical School, Southmead Hospital, Bristol, UK

Email: t.j.fowler@bristol.ac.uk; Phone: +44 (0)117 414 7865

| **Reply to comments: “Association between surgeon training grade and the risk of revision following total knee replacement: an analysis of a National Joint Registry Data” (PMEDICINE-D-24-04032R3)** | | |
| --- | --- | --- |
|  | | |
| **Reviewer & Comment number** | **Comment** | **Response** |
| **Reviewer 1: Comment 1** | *Thank you I am satisfied with your responses.* | Thank you again for taking the time to review our paper. We are grateful for your input. |
| **Reviewer 2: Comment 1** | *We thank the authors for their detailed responses, and commitment to release related code upon publication. Nonetheless, regarding the diagnostic criteria for all-cause revision, some additional detail on such might be appropriate in the supplementary material. In particular, the effect of follow-up consultation frequency, and any cost/insurance/functional quality-of-life considerations (if relevant) might be discussed as to whether and to what extent they might affect a revision operation taking place.* | Thank you again for taking the time to review our paper. We are grateful for your input and for your recommendation. We have written the following response, which we have included at the end of the supplementary materials:  *“Supplementary Note: Considerations Influencing All-Cause Revision.*  *Please note, the following supplementary discussion has been added in response to a comment raised by one of the reviewers during the peer-review process.*  *The primary outcome of this study was all-cause revision, defined by the NJR as “any procedure to add, remove, or modify one or more components of an implant construct for any reason.”*  *While the NJR robustly captures revision procedures across England and Wales, several external factors may influence whether and when a revision occurs. These include:*   - ***Follow-up consultation practices****: There is variability in post-operative follow-up across institutions and over time, particularly in how routine follow-up is structured beyond the first year. However, patients with concerning symptoms or functional decline are typically re-referred to orthopaedic services via primary care, and most revisions are captured regardless of formal follow-up arrangements.* - ***Healthcare funding and cost considerations****: As the UK’s National Health Service (NHS) provides care free at the point of delivery, direct patient costs or insurance status do not influence revision decisions. This differs from other healthcare systems and reduces potential financial barriers to revision surgery.* - ***Patient-reported outcomes and quality of life****: Although PROMs and functional scores may influence a clinician’s decision to revise, they are not routinely linked to NJR data. Therefore, we were not able to assess the association between PROMs and episodes of revision surgery in the context of surgical training.*   *In summary, while we acknowledge that individual clinical decisions around revision are complex and multifactorial, the use of all-cause revision within the NJR represents a consistently applied, objective, and widely accepted outcome measure in joint replacement research.”* |
| **Reviewer 3:**  **Comment 1** | *My comments have been addressed adequately given the limitations of the data available in the NJR. The lack of ability to provide a better analysis that could influence future training and hence improve patient outcomes is disappointing. However, it is an important subject and the finding of adequate revision rates from trainee led TKRs is helpful. The discussion covers the limitations appropriately.* | Thank you again for taking the time to review our paper. We are grateful for your input. |
| **General Editorial Request: 1** | *Please confirm that your title complies with to PLOS Medicine's style. Your title must be nondeclarative and not a question. It should begin with Statistical reporting: Please revise throughout the manuscript, including tables and figures. - Please report statistical information as follows to improve clarity for the reader ""22% (95% CI [13,28]; p</=)"". - Please separate upper and lower bounds with commas instead of hyphens as the latter can be confused with reporting of negative values. - Please repeat statistical definitions (HR, CI etc.) for each set of parentheses.main concept if possible. "Effect of" should be used only if causality can be inferred, i.e., for an RCT. Please place the study design ("A randomized controlled trial," "A retrospective study," "A modelling study," etc.) in the subtitle (i.e., after a colon).* | We can confirm that our title complies with PLOS Medicine’s style. We have edited it slightly by removing the first word ‘The’ to make it consistent with our previous paper on a related subject, which is published in PLOS Medicine. <https://doi.org/10.1371/journal.pmed.1004445>  We have edited the manuscript throughout to report 95% CIs as requested.  We have defined hazard ratio (HR) and confidence interval (CI) at first use. |
| **General Editorial Request: 2** | *Please ensure that all abbreviations are defined at first use throughout the text (including statistical abbreviations). Please also check figures and tables.* | We have checked the text and tables to ensure that all abbreviations are defined at first use. |
| **General Editorial Request: 3** | *Please ensure that tables and figures, including those in supplementary files, are appropriately referenced in the main text.* | We have checked the text and supplementary files to ensure that figures are appropriately referenced. |
| **General Editorial Request: 4** | *Financial Disclosure: If available, please provide URL(s) of each funder website.* | Not available. |
| **General Editorial Request: 5** | *Please check that any use of statistical terms (such as trend or significant) are supported by the data, and if not please remove them.* | Any use of these terms is supported by the data. The use of such terms in minimal in this paper. |
| **General Editorial Request: 6** | *You have stated that you are happy to make author-generated code for this paper available on publication. Please add a sentence to your data availability statement regarding any code used in the study, e.g. "The code used in the analysis is available from Github [URL] and archived in Zenodo [DOI link]" Please ensure that your code is shared in a way that follows best practice and facilitates reproducibility and reuse.* | We have uploaded the author generated code to Github and Zenodo and included the recommended statement in our data availability statement as follows:  *“The code used in the analysis is available from Github [https://github.com/tjfowler89/Stata_Code_Fowler/tree/main] and archived in Zenodo [https://doi.org/10.5281/zenodo.15785216].”* |
| **General Editorial Request: 7** | *ABSTRACT: Please confirm that your abstract complies with our requirements, including providing all the information relevant to this study type* [*https://journals.plos.org/plosmedicine/s/submission-guidelines#loc-abstract*](https://journals.plos.org/plosmedicine/s/submission-guidelines#loc-abstract) | We confirm that the abstract complies with the PLOS requirements. |
| **General Editorial Request: 8** | *ABSTRACT: Please ensure that all numbers presented in the abstract are present and identical to numbers presented in the main manuscript text.* | We confirm this. |
| **General Editorial Request: 9** | *ABSTRACT: If the data is available, we think it would be helpful to mention the number of trainees and consultants you assessed.* | The data for this are not available. Please see our explanation for this in our previous detailed response.  *“Unfortunately, this is not feasible with current NJR data. While some trainee-performed cases are recorded using a unique Surgeon ID that would enable longitudinal tracking of an individual trainee’s practice, in many instances, trainee cases are instead recorded under the supervising consultant’s ID. This inconsistency in how trainee procedures are attributed means it is not possible to reliably identify and follow individual trainees through to their later consultant practice.”*  For this reason we are unable to state the exact number of trainees assessed. |
| **General Editorial Request: 10** | *ABSTRACT:* *Given our international readership, we believe it would be useful to provide the average number of years of experience for trainees and consultants.* | Again, for the same reason explained above, it is not possible to accurately calculate the average number of years of experience for individual surgeons/trainees. However, we have explained this in detail in the ‘Exposures’ subsection of the Methods section and even included a detailed flow diagram in the supplementary materials (S3 Appendix) to further illustrate this for international readers. The flow diagram shows the approximate years of experience for trainees at each stage in training.  We have taken time to discuss and illustrate the differences between trainees and consultants and clarified international equivalents. We feel this is too large a topic to discuss in detail in the abstract, but it is covered in detail in the main paper.  In response to your request, we have added the following to the Abstract: *“Consultant surgeons have been awarded a Certificate of Completion of Training (CCT) in orthopaedic surgery and have been appointed to a senior position in which they can supervise trainees. The term ‘consultant’ is synonymous with ‘attending’ in several healthcare settings including the USA.”* |
| **General Editorial Request: 11** | *ABSTRACT: Please quantify the main results (with 95% CIs and p values).* | The main results are displayed in hazard ratio (HR) plots. Thus descriptive terms are used in the abstract. Due to the time-dependent nature of the data, it is not appropriate to quantify the main results in these terms. Please see our explanation of this in the methods section. |
| **General Editorial Request: 12** | *ABSTRACT: Your study is observational and therefore causality cannot be inferred. Please remove language that implies causality, such as effect. Refer to associations instead. Please revise throughout the main text.* | We have revised this throughout the manuscript and swapped the word effect for association in numerous circumstances.  In the context of FPM models, we have continued to refer to “time-dependent effects” as this is the terminology used by Royston & Lambert or this form of statistical methodology. We are not claiming causal effect in these statements. |
| **General Editorial Request: 13** | *AUTHOR SUMMARY: If you prefer, you may write the author summary from a first-person plural perspective, i.e., "we."* | We have made this change throughout the Author Summary. |
| **General Editorial Request: 14** | *INTRODUCTION: If you agree, we suggest defining the ‘specific indications’ in the last sentence.* | We have added the list of indications to the last sentence. |
| **General Editorial Request: 15** | *METHODS AND RESULTS: You stated that the study protocol was planned in detail prior to commencing the study, yet you also said that such a document does not exist. Please clarify. If the document exists and was used to design the study, include it as a Supporting Information file in your revised manuscript to be published alongside your study, and cite it in the Methods section. If the document does not exist, revise the statement in the main text for clarity.* | The study protocol was designed prior to commencing this study including the study population, exposures, outcomes of interest and statistical methods. You will note that the protocol and analsyses used throughout this paper are comparable to those used in our related publications on the outcomes of trainee-performed joint replacement, which pre-date this study:   - <https://doi.org/10.1371/journal.pmed.1004445> - <https://doi.org/10.1302/0301-620X.104B3.BJJ-2021-1389.R1>   Furthermore, we have documented a summary of the process of model selection/construction in S2 Appendix. However, the document that you are requesting does not exist. Therefore, according to your recommendation we have deleted the short “Analysis plan” paragraph. |
| **General Editorial Request: 16** | *METHODS AND RESULTS: Do you have data on how many trainees and consultants performed TKRs? If so, we think it would be valuable to include these.* | The data for this are not available. Please see our explanation for this in our previous detailed response.  *“Unfortunately, this is not feasible with current NJR data. While some trainee-performed cases are recorded using a unique Surgeon ID that would enable longitudinal tracking of an individual trainee’s practice, in many instances, trainee cases are instead recorded under the supervising consultant’s ID. This inconsistency in how trainee procedures are attributed means it is not possible to reliably identify and follow individual trainees through to their later consultant practice.”*  For this reason we are unable to state the exact number of trainees/consultants assessed. |
| **General Editorial Request: 17** | *METHODS AND RESULTS: We have noted that you switch between the descriptions “all-cause failure” and “all-cause revision” for the primary outcome. We recommend using the same term consistently.* | We have standardised this throughout the manuscript to “all-cause revision”. |
| **General Editorial Request: 18** | *METHODS AND RESULTS: You discuss marginal differences between trainee and consultant outcomes multiple times. Given these small statistical differences, we think it’s important to discuss the clinical relevance of these findings. We have highlighted some of the relevant statements below: - “There is a subtle divergence in the probability of failure between 1 and 4 years, with separation between the confidence intervals noted at the 3-year interval.” - “Despite extensive adjustment for confounding factors, the marginal association between trainee-performed TKR and risk of revision in the first 4 years remains consistent.” - Fully adjusted models are displayed in Figure 7 and indicate marginal associations between trainee-performed TKR and early revision for aseptic loosening/lysis (up to 3 years), infection (up to 3 years), and progression of OA (up to 5 years).* | We thank the editors for this helpful suggestion. While we believe that we have already discussed the clinical relevance of our findings in appropriate manner in the discussion, we acknowledge that further clarification may be warranted.  Although our adjusted models identified small increases in early revision risk for specific indications, the absolute differences were minimal. It is important to interpret these findings alongside the unadjusted analyses, which are particularly reassuring and show that implant survivorship for trainee-performed TKRs is comparable to that of consultant-performed procedures. In response to your request we have now added the following paragraph to the discussion (second to last paragraph):  *“While our adjusted analyses identified small differences in early revision risk between consultant- and trainee-performed TKRs, the absolute differences were minimal. These marginal associations are unlikely to be of clinical significance, particularly when interpreted alongside our unadjusted analyses, which show that overall implant survival remains high and comparable across both groups. Taken together, these findings are reassuring and support the safety of trainee involvement in TKR, provided that appropriate case selection and supervision are in place.”* |
| **General Editorial Request: 19** | *METHODS AND RESULTS: “Surgeon grade had a significant time-dependent effect” – We feel this is an overstatement and the term ‘effect’ should only be used if causality can be inferred.* | We have edited this to now read “time-dependent association”. |
| **General Editorial Request: 20** | *Figure 3/4/5/6: Please provide a more specific title for the y-axis.* | We have reviewed the y-axis for each of these Figures. These are all hazard ratio plots and the y-axis in each case is correctly labelled as the “Hazard ratio”. It would not be accurate to label this otherwise. However, we have provided detailed figure legends to go under each figure, which add further information for the reader and aid in interpretation. Please see this on page 24.  We give the following explanation for Figure 3, and for clarity, we have now added a similar statement to the legend for figures 4-7: *“The solid black line represents the HR, and the shaded grey area represents the 95% CI. Separation between the upper or lower 95% CI and the baseline of 1.00 (representing consultant-performed procedures) is suggestive of an association.”*  We hope you agree that this adds clarity, but also prevents overcomplication/mislabelling of the y-axis. |
| **General Editorial Request: 21** | *Figure 1: Please define all abbreviations used in the figure.* | All abbreviations have now been defined as requested. These are included in the Figure file. |
| **General Editorial Request: 22** | *Figure 3: For the two graphs depicting the results of the sensitivity analyses, please include the numbers of cases included.* | Case numbers have now been included in the figure legends, which should go under each figure, on Page 24. |
| **General Editorial Request: 23** | *Figure 4/6: Please include case numbers.* | Case numbers have now been included in the figure legends, which should go under each figure, on Page 24. |
| **General Editorial Request: 24** | *Figure 7: Please ensure that the y-axis is identical for all figures to facilitate comparison.* | We have now edited Figure 7 to ensure all graphs are on an identical y-axis. |
| **General Editorial Request: 25** | *DISCUSSION:* *Please review your text for claims of novelty or primacy (e.g. 'for the first time') and remove this language.* | The word novel has been removed from the first line of the discussion. |
| **General Editorial Request: 26** | *DISCUSSION: Please remove all subheadings.* | All subheadings have been removed |
| **General Editorial Request: 27** | *We ask every co-author listed on the manuscript to fill in a contributing author statement. If any of the co-authors have not filled in the statement, we will remind them to do so when the paper is revised. If all statements are not completed in a timely fashion this could hold up the re-review process. Should there be a problem getting one of your co-authors to fill in a statement we will be in contact. YOU MUST NOT ADD OR REMOVE AUTHORS UNLESS YOU HAVE ALERTED THE EDITOR HANDLING THE MANUSCRIPT TO THE CHANGE AND THEY SPECIFICALLY HAVE AGREED TO IT.* | All authors have completed a contributing author statement. |
| **General Editorial Request: 28** | *Please ensure that the paper adheres to the PLOS Data Availability Policy (see* [*http://journals.plos.org/plosmedicine/s/data-availability)*](http://journals.plos.org/plosmedicine/s/data-availability))*, which requires that all data underlying the study's findings be provided in a repository or as Supporting Information. For data residing with a third party, authors are required to provide instructions with contact information for obtaining the data. PLOS journals do not allow statements supported by "data not shown" or "unpublished results." For such statements, authors must provide supporting data or cite public sources that include it.* | Please see our data availability statement:  “The data used in the study are available from The National Joint Registry (NJR) (https://www.njrcentre.org.uk). Restrictions apply to the availability of these data, which were used under license for the current study, and are therefore not publicly available. Data access applications can be made to the NJR Research Committee. With NJR permission in place, the data underlying the presented results will be available to access via the NJR data access network. The authors of this manuscript are not the data owner and do not have permission to share the data.” |
| **General Editorial Request: 29** | *Please review your reference list to ensure that it is complete and correct. If you have cited papers that have been retracted, please include the rationale for doing so in the manuscript text, or remove these references and replace them with relevant current references. Any changes to the reference list should be mentioned in the rebuttal letter that accompanies your revised manuscript.* | We have reviewed the reference list, which is complete and correct. |
