# Supplementary material for: Association between surgeon training grade and the risk of revision following total knee replacement: An analysis of National Joint Registry data
Source: PLoS Med. 2025 Aug 12;22(8):e1004685. doi: 10.1371/journal.pmed.1004685 (PMC12370202; doi:10.1371/journal.pmed.1004685)
Supplement: Attachment — Submitted filename: Response_auresp_4.docx [file pmed.1004685.s015.docx]

Mr T J Fowler

Musculoskeletal Research Unit

Translational Health Sciences

Bristol Medical School

Southmead Hospital, Bristol

United Kingdom

**Prof. Dr. Till Bärnighausen**

Editor-in-Chief

PLOS Medicine

July 15th, 2025

Dear **Prof. Dr. Bärnighausen**,

**Reply to comments: “Association between surgeon training grade and the risk of revision following total knee replacement: an analysis of a National Joint Registry Data” (PMEDICINE-D-24-04032R3)**

Thank you again for taking the time to review our paper and for the detailed appraisal of our work. Once again, we would like to thank the editorial team for their time in re-reviewing our work. I am pleased to attach our re-revised article, as well as our response to each of the editorial comments. We have addressed all of the comments and have edited the manuscript accordingly. We hope that you find these changes satisfactory. Please note that we have swapped S2 Appendix and S3 Appendix, so that they are now referenced in the correct order.

Yours sincerely,

Mr Tim Fowler

BM BCh (Oxon) MA MD FRCS

Academic Clinical Fellow & Orthopaedic Registrar

Musculoskeletal Research Unit, Bristol Medical School, Southmead Hospital, Bristol, UK

Email: t.j.fowler@bristol.ac.uk; Phone: +44 (0)117 414 7865

| **Reply to comments: “Association between surgeon training grade and the risk of revision following total knee replacement: an analysis of a National Joint Registry Data” (PMEDICINE-D-24-04032R3)** | | |
| --- | --- | --- |
|  | | |
| **Reviewer & Comment number** | **Comment** | **Response** |
| **General Editorial Request: 1** | *In the Abstract Background, please explain “revision”. For example: Revision surgery is any subsequent surgery performed to correct or improve the results of an earlier procedure.* | Thank you for your recommendation. We have now added the following to the background subsection of the abstract:  *“Revision is defined by the National Joint Registry for England and Wales (NJR) as any procedure to add, remove, or modify one or more components of an implant construct for any reason.”* |
| **General Editorial Request: 2** | *In the Abstract, please include the important dependent variables that are adjusted for in the analyses (briefly explain Model 1, 2, 3 and 4).* | We have now edited this section of the abstract to read as follows:  *“Flexible parametric survival models (FPM) were incrementally adjusted in the following manner. Model 1 was unadjusted. Model 2 was adjusted for patient-level factors (age, sex, American Society of Anaesthesiologists [ASA] grade, and index of multiple deprivation [IMD] decile). Model 3 was further adjusted for operation-level factors (anaesthetic, approach, fixation, constraint and whether or not the patella was resurfaced). Model 4 was further adjusted for healthcare setting factors (funding source, and year of operation).”* |
| **General Editorial Request: 3** | *Abstract: Thank you for your response to our request for the average number of years of experience for trainees and consultants. Upon further consideration, we suggest removing the text.* | We have now deleted this sentence from the abstract as requested. |
| **General Editorial Request: 4** | *While we understand that your analysis presents data in a time-dependent nature, we are convinced that the inclusion of numerical results is essential for accurate reporting. Please note that we have some leeway regarding Abstract length for the sake of reporting transparency. We have outlined two suggestions below: - Suggestion #1: “**Trainees achieved comparable outcomes to consultants in terms of the unadjusted cumulative probability of all-cause revision (e.g. 5 years of follow-up: consultant % Failure 2.21 (95% CI [2.17, 2.24]) versus trainee (overall) % Failure 2.33 (95% CI [2.22, 2.44])).” - Suggestion #2: “Adjusted FPM analysis indicated evidence of an association between trainee-performed TKR and a small increased risk of early all cause revision up to, but not exceeding, 4 years follow-up (1 year: HR 1.12 (95% CI [1.05, 1.19]), 4 years: HR 1.00 (95% CI [0.95, 1.06]), 16 years: HR 0.89 (95% CI [0.81, 0.98])).”* | Thank your careful consideration here and for your recommendation. We have now edited this accordingly and included your Suggestion #1. Please note that we have used the 15-year interval of follow-up instead of 5-years, which is consistent with the interval used in the abstract of our previous related paper in UKRs (<https://doi.org/10.1371/journal.pmed.1004445>). This reads as follows:  “Trainees achieved comparable outcomes to consultants in terms of the unadjusted cumulative probability of all-cause revision *(e.g. 15 years of follow-up: consultant % Failure 4.79 (95% CI [4.67, 4.92]) versus trainee (overall) % Failure 4.75 (95% CI [4.43, 5.10])*.” |
| **General Editorial Request: 5** | *For Figure 3-7, please provide the hazard ratios and CIs in tabular format as a Supporting Information file.* | Thank you for this suggestion and for the additional time that was granted to complete this request. We have now included HRs and CIs in tabular format within the Supporting Information file. A separate table has been provided for each of Figures 3-7. This is referred to as S5 Appendix within the text. We have used the following intervals of follow-up: 1, 3, 5, 7, 10, 13, 15 years. This is consistent with the time intervals in Table 2, which are the ODEP (Orthopaedic Device Evaluation Panel) standard intervals of follow-up. |
| **General Editorial Request: 6** | *“Surgeon grade had a significant time-dependent effect” (l.527 track changes) – Please note that although you said you adjusted the statement, it remains unrevised.* | Please excuse this omission. We have now revised this sentence, which reads:  *“Surgeon grade had a significant time-dependent association, and it is an oversimplification to report single numeric hazard ratio (HR).”* |
| **General Editorial Request: 7** | *S3 Figure: In the Kaplan-Meier curve(s) please provide the number at risk for each time interval.* | Thank you for this suggestion. We have now provided number at risk tables for each KM plot in S3 Figure. |
